# Serotype-Dependent Inhibition of *Streptococcus pneumoniae* Growth by Short-Chain Fatty Acids

**DOI:** 10.4014/jmb.2309.09003

**Published:** 2023-11-20

**Authors:** Suwon Lim, Dongwook Lee, Sungho Jeong, Jeong Woo Park, Jintaek Im, Bokeum Choi, Donghyun Gwak, Cheol-Heui Yun, Ho Seong Seo, Seung Hyun Han

**Affiliations:** 1 Department of Oral Microbiology Immunology, Dental Research Institute, School of Dentistry, Seoul National University, Seoul 08826, Republic of Korea; 2 Department of Agricultural Biotechnology, and Research Institute of Agriculture and Life Sciences, Seoul National University, Seoul 08826, Republic of Korea; 3 Institutes of Green Bio Science and Technology, Seoul National University, Pyeongchang 25354, Republic of Korea; 4 Research Division for Biotechnology, Korea Atomic Energy Research Institute, Jeongeup 56212, Republic of Korea

**Keywords:** *Streptococcus pneumoniae*, short-chain fatty acids, antimicrobial agent, serotypes

## Abstract

*Streptococcus pneumoniae* (pneumococcus) is an opportunistic pathogen that can cause severe infectious diseases such as pneumonia, meningitis, and otitis media. Despite the availability of antibiotics and pneumococcal vaccines against some invasive serotypes, pneumococcal infection remains a tremendous clinical challenge due to the increasing frequency of infection by antimicrobial resistant, nonencapsulated, and/or non-vaccine serotype strains. Short-chain fatty acids (SCFAs), which are produced at various mucosal sites in the body, have potent antimicrobial activity, including inhibition of pathogen growth and/or bacterial biofilm formation. In this study, we investigated the antimicrobial activity of SCFAs (acetate, propionate, and butyrate) against various serotypes pneumococci. Propionate generally inhibited the growth of *S. pneumoniae* serotypes included in the pneumococcal conjugate vaccine (PCV) 13, except for serotypes 3 and 7F, though butyrate and acetate showed no or low inhibition, depending on the serotypes. Of note, butyrate showed strong inhibition against serotype 3, the most prevalent invasive strain since the introduction of the PCV. No SCFAs showed inhibitory effects against serotype 7F. Remarkably, the nonencapsulated pneumococcal strain had more sensitivity to SCFAs than encapsulated parental strains. Taken together, these results suggest that propionate showing the most potent inhibition of pneumococcal growth may be used as an alternative treatment for pneumococcal infection, and that butyrate could be used against serotype 3, which is becoming a serious threat.

## Introduction

*Streptococcus pneumoniae* (pneumococcus) is a facultative anaerobic Gram-positive bacterium that asympto-matically colonizes the upper respiratory tract. *S. pneumoniae* can cause serious invasive diseases such as pneumonia, bacteremia, and meningitis [1]. The polysaccharide capsule is a dominant surface structure of *S. pneumoniae* and plays a critical role in virulence, facilitating nasopharyngeal colonization and infection by antibody-dependent opsonophagocytic clearance mechanisms [2]. Approximately 100 serotypes are classified according to the structure of capsular polysaccharide (CPS) and each serotype induces a unique serologic response leading to the production of specific antibodies [3]. Accordingly, vaccines such as pneumococcal polysaccharide vaccine (PPSV) and pneumococcal conjugate vaccine (PCV) have been developed to target CPS of *S. pneumoniae* [4].

Despite the availability of vaccines against major serotypes associated with invasive pneumococcal diseases (IPDs), in 2020, IPD still resulted in 5.4 cases with 0.78 deaths per 100,000 persons in the United States [5]. According to the previous study, the persistent pneumococcal infections were mainly caused by serotypes 19A and 3 which belong to vaccine serotypes, and newly emerging non-vaccine serotypes 12F and 23B [6]. In addition, nonencapsulated pneumococci are consistently found in asymptomatic carriage since the introduction of PCV [7]. Furthermore, pneumococci readily acquire antibiotic resistance, including resistance to penicillin, macrolides, and fluoroquinolones [8]. Therefore, pneumococcal infection remains a tremendous clinical challenge, and several alternative therapeutic strategies are being investigated. Kaempferol, a natural flavonoid, attenuated pneumococcal lung infection in a mouse pneumonia model and clofilium has been reported as a novel antibacterial compound that inhibits pneumococcal growth and viability by permeabilizing the cell membrane [9, 10].

Recently, there has been increasing interest in the potential use of short-chain fatty acids (SCFAs) as antimicrobial agents [11]. SCFAs, mainly composed of acetate, propionate, and butyrate, are produced by commensal bacteria at various mucosal sites, including gut, oral cavity, and lung [12-14]. They play a variety of physiological roles, such as being the primary energy source of colonocytes, regulators of glucose or lipid biosynthesis, and modulators of inflammation and immunity [15-17]. In addition, SCFAs have anti-microbial activity that prevents the colonization of pathogenic bacteria by inhibiting their growth and/or biofilm formation. For instance, SCFAs reduced the growth of *Salmonella* Typhimurim by more than 50% and interfered with its motility and biofilm formation [18, 19]. Propionate effectively inhibited the growth of *Staphylococcus aureus* and *Enterococcus faecalis* [20] and suppressed the biofilm formation of *Streptococcus gordonii* without affecting its growth [21].

In this study, we investigated the antimicrobial activity of SCFAs on *S. pneumoniae* and determined their relative activities against 13 capsular serotype pneumococci and capsule-deficient pneumococci to explore whether SCFAs have broad spectrum anti-pneumococcal activity.

## Materials and Methods

### Reagents and Chemicals

Sodium acetate (NaA), sodium propionate (NaP), and sodium butyrate (NaB) were purchased from Sigma-Aldrich (USA). These SCFAs were dissolved in endotoxin-free water (Dai Han Pharm Inc., Korea) and filtered with a 0.2 μm syringe filter (Corning, USA). Defibrinated sheep blood was purchased from Kisan Bio (Korea). Tryptic soy broth, Todd-Hewitt broth, Bacto agar, and yeast extract were purchased from BD Biosciences (USA).

### Bacterial Strains and Culture Conditions

*S. pneumoniae* serotype 1 (NCCP15882) was purchased from the National Culture Collection for Pathogens (Korea). *S. pneumoniae* serotype 3 (ATCC 6303), TIGR4 (ATCC BAA-334), 19F (ATCC 49619), and R36A (ATCC 27336) were purchased from American Type Culture Collection (USA). Serotypes 5, 6A, 6B, 7F, 9V, 14, 18C, 19A, 23F, and R6 pneumococci, were kindly provided by Prof. Moon H. Nahm at the University of Alabama at Birmingham (USA). All pneumococcal strains were grown on blood agar plates or in Todd-Hewitt broth with 0.5% yeast extract (THY) at an anaerobic workstation (Whitley DG250, Don Whitley Scientific, UK) maintaining 10% CO_2_ at 37°C.

### Generation of CPS-deficient TIGR4

A CPS-deficient TIGR4 mutant (Δ*cps*) was produced as previously described [22]. Briefly, an upstream region of *cps2B* was amplified by using primers Cps2KO-UpF (5’-AAC TCG AGT GGA TAT CAA TTA CTA T-3’) and Cps2BKO-UpR (5’-TTA AGC TTT CAT CTA CCC TCC ATC-3’) and cleaved by *XhoI* and *Hind*III. A downstream region of *cps2B* gene was amplified with primers, Cps2CKO-DnF (5’-AAG AAT TCT GGT AAA AGA CTA CCG TG-3’) and Cps2CKO-DnR (5’-TTG AAT TCT ATT TCA ACT TAC CCA AG-3’) and cleaved by EcoRI. Then, PCR products were inserted into multicloning site of pE326 [23]. pKO-*cps2B* gene, the plasmid inserted PCR product, was transformed into TIGR4 by natural transformation [24]. Deletion of CPS was confirmed by enzyme-linked immunosorbent assay (ELISA) as previously described [25, 26].

### Measurement of Growth and Susceptibility of *S. pneumoniae*

Measurement of pneumococcal growth was conducted as previously described with slight modifications [20]. Two percent pneumococcal seed culture was inoculated to fresh THY with the indicated concentrations of SCFAs in uncoated flat bottom 96-well plates (SPL Life Sciences, Korea) under anaerobic condition. The growth was measured at each time point at 600 nm of optical density (OD_600_) by spectrophotometer (SPARK, Molecular Devices, USA). The susceptibility was calculated as the OD_600_ value of the 100 mM SCFAs treated group divided by the OD_600_ value of the non-treated group (NT) at the maximum growth time.

### Minimum Inhibitory Concentration (MIC)

To determine the MIC of SCFAs, two percent of the seed culture was inoculated in the fresh medium containing various doses (0, 3.9, 7.8, 15.6, 31.3, 62.5, 125, 500, or 1,000 mM) of SCFAs and incubated for 12 h. The minimum concentration at which the bacterial growth does not occur was determined by comparing to wells that were not treated with SCFAs.

### Statistical Analysis

The data in all figures represent at least three independent experiments and indicates the mean value ± standard deviation of triplicate samples. Statistical significance was determined with Student’s *t*-test by comparing with the non-treatment (NT) group, wild type (WT), and encapsulated strain. *P*-values less than or equal to 0.05 are indicated by asterisks (*).

## Results

### SCFAs Inhibit *S. pneumoniae* TIGR4 Growth

**Fig. 1:**
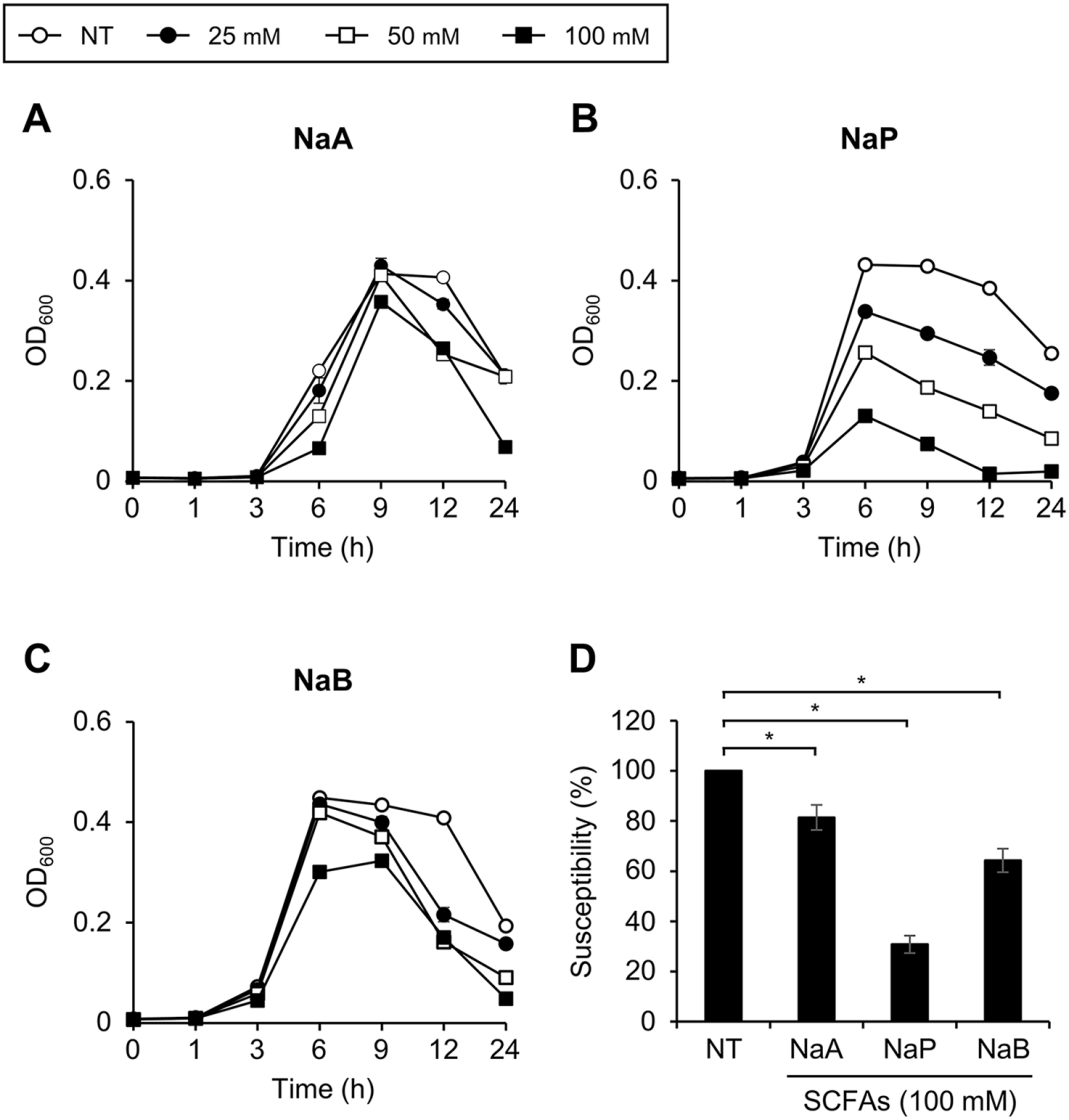
Growth inhibition of *S. pneumoniae* TIGR4 by SCFAs. *S. pneumoniae* TIGR4 (serotype 4) was cultured in media with or without various doses of (**A**) NaA, (**B**) NaP, and (**C**) NaB. The OD_600_ value was measured at 0, 1, 3, 6, 9, 12, or 24 h. (**D**) At the maximum growth time point of NT, the susceptibility is calculated as OD_600_ value of 100 mM SCFAs treated group divided by maximum OD_600_ value of NT. Data shown are representative of three independent experiments and indicate the mean value ± standard deviation of triplicate samples. Statistical significance was analyzed with Student’s *t*-test compared to NT or among SCFAs treated groups (*, *P* < 0.05). NaA, sodium acetate; NaP, sodium propionate; NaB, sodium butyrate; NT, non-treatment.

We initially examined whether SCFAs could inhibit the growth of *S. pneumoniae*, the most common pathogenic bacteria colonizing the nasal mucosa. As shown in Fig. 1A-1C, the growth of *S. pneumoniae* TIGR4 (serotype 4) was significantly inhibited in a dose-dependent manner in propionate medium, but only modest inhibition was found at high concentrations (100 mM) of acetate and butyrate media. To determine the relative inhibition of each SCFA, we compared the maximum OD_600_ of TIGR4 in medium with 100 mM of each SCFA (Fig. 1D). While all SCFAs tested were found to significantly inhibit TIGR4 growth, propionate was found to be the most significant inhibitor over acetate and butyrate.

### Growth Inhibition by SCFAs Displays Serotype-Dependency

**Fig. 2:**
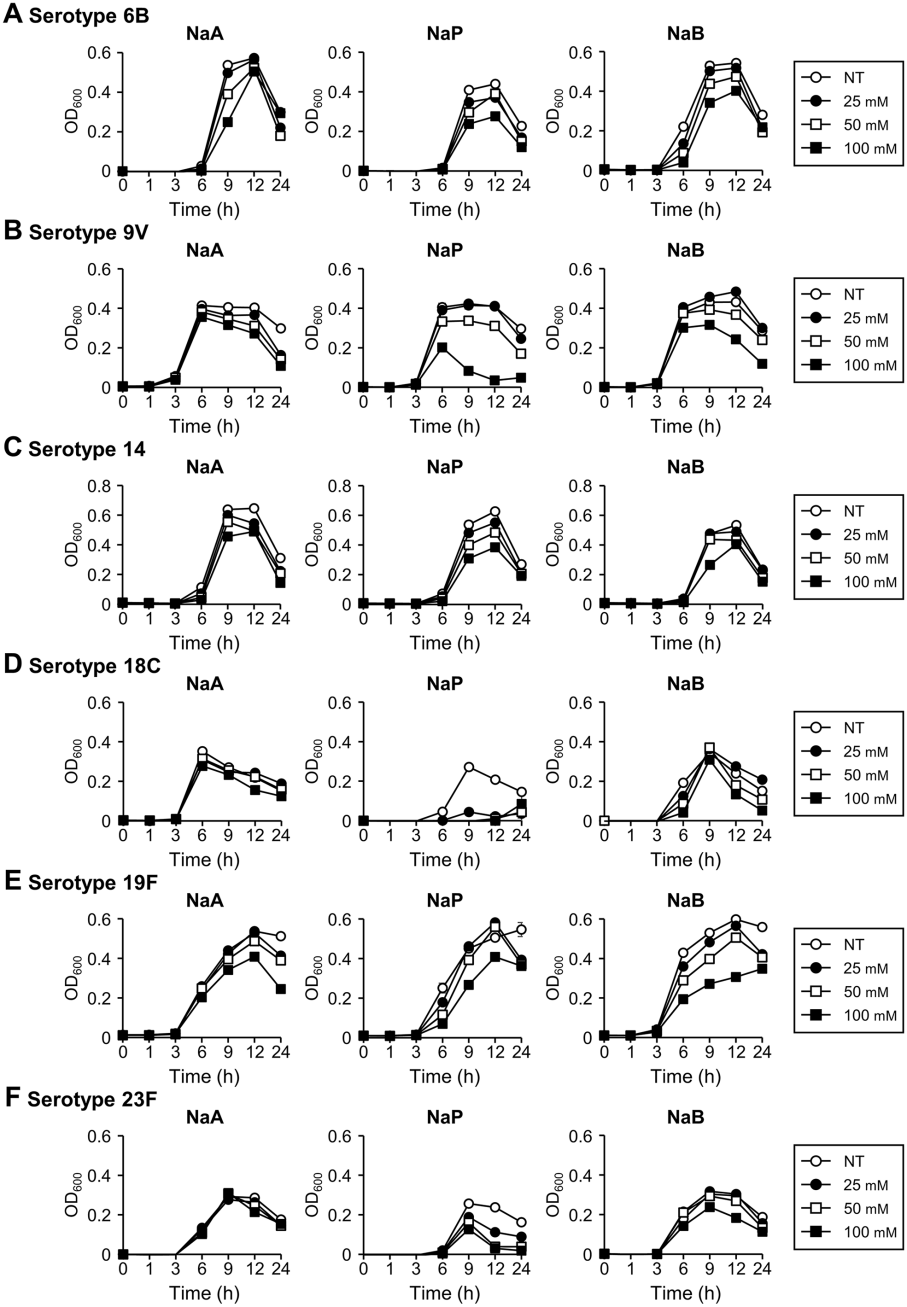
Growth inhibition of PCV7 serotypes by SCFAs. *S. pneumoniae* serotypes (**A**) 6B, (**B**) 9V, (**C**) 14, (**D**) 18C, (**E**) 19F, and (**F**) 23F were cultured in media with 25, 50 or 100 mM or without SCFAs. The OD_600_ value was measured at 0, 1, 3, 6, 9, 12, or 24 h. The data displayed represent the average value with standard deviation of triplicate samples from three separate experiments. NaA, sodium acetate; NaP, sodium propionate; NaB, sodium butyrate; NT, non-treatment.

To investigate whether the inhibition of pneumococcal growth by SCFAs is general or serotype-dependent, we first analyzed its growth inhibition against the most prevalent serotypes included in Prevnar 7 vaccine (6B, 9V, 14, 18C, 19F, and 23F) inasmuch as TIGR4 (serotype 4) tested in Fig. 1A-1D which also belongs to the vaccine serotypes. As shown in Fig. 2A-2F, we found that the growth inhibition of SCFAs for each serotype was distinct. Acetate had a strong inhibitory effect against 19F, but no significant inhibition was found for other serotypes. Propionate was the most effective growth inhibitor with dramatic inhibition for serotypes 9V, 18C, and 23F and modest inhibition for serotypes 6B, 14, and 19F. For butyrate, a dramatic inhibition was seen for 6B, 9V, and 19F though no significant inhibitory effect on serotype 4 (TIGR4) was found above in Fig. 1A-1D.

**Fig. 3:**
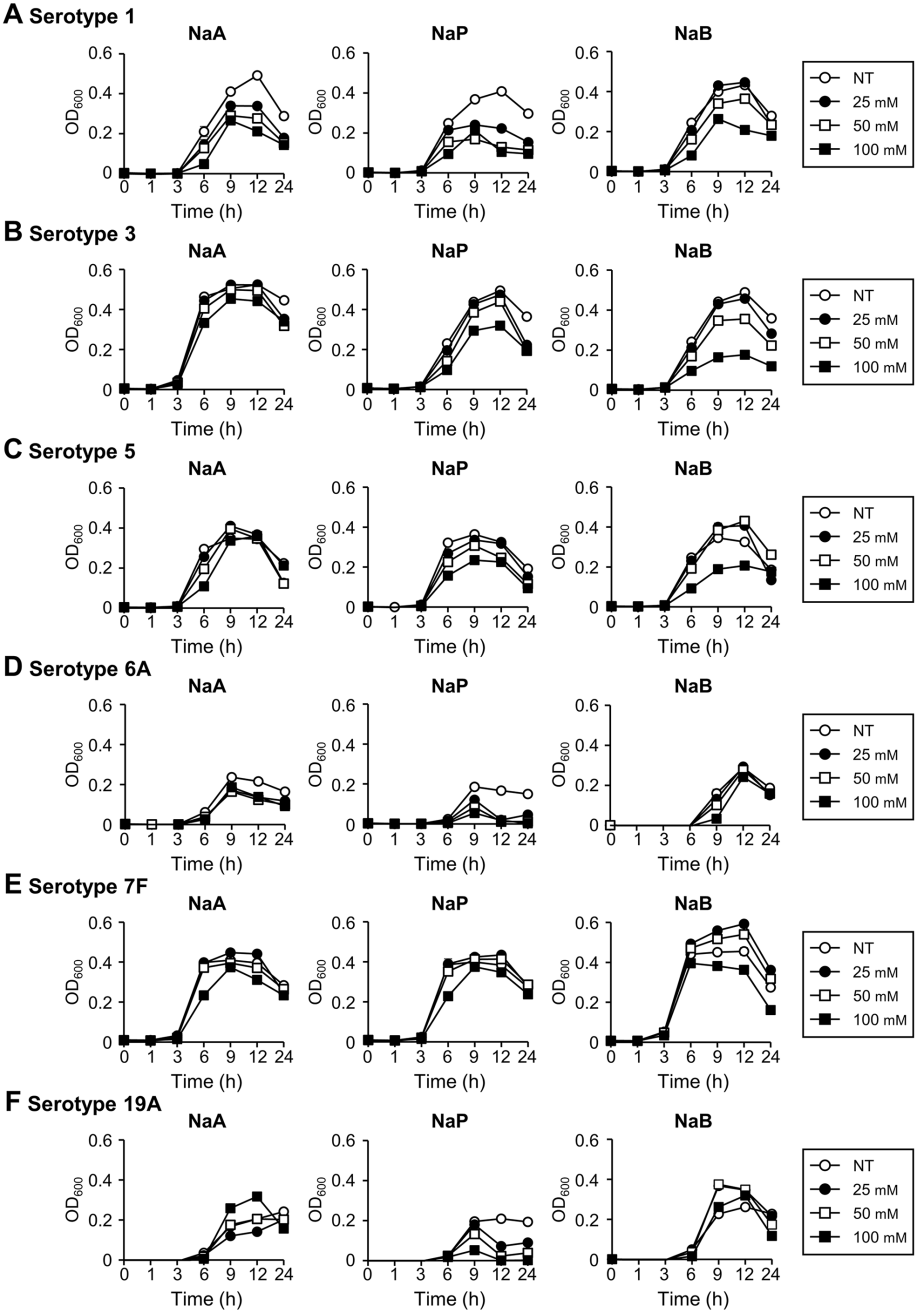
Growth inhibition of PCV13 serotypes except PCV7 serotypes. *S. pneumoniae* serotypes (**A**) 1, (**B**) 3, (**C**) 5, (**D**) 6A, (**E**) 7F, and (**F**) 19A were cultured in media with 25, 50 or 100 mM or without SCFAs. The OD_600_ value was measured at 0, 1, 3, 6, 9, 12, or 24 h. The data displayed represent three different experiments and represent the mean value and standard deviation of triplicate samples. NaA, sodium acetate; NaP, sodium propionate; NaB, sodium butyrate; NT, non-treatment.

Serotype-dependent growth inhibition of pneumococcus by SCFAs was confirmed for six additional serotypes, which are included in Prevnar 13 (serotypes of Prevnar 7 plus 1, 3, 5, 6A, 7F, and 19A serotypes) and account for approximately 37.4% of all invasive pneumococcal disease in countries where PCV10 and PCV13 were introduced [27]. As shown in Fig. 3A-3F, acetate showed the most potent inhibitory effect against serotype 1, but no significant inhibition was found for the other serotypes. Propionate most effectively inhibited the growth of serotypes 1, 6A, and 19A. Butyrate had dramatic inhibition against serotypes 1, 3, and 5 and modest inhibition on serotype 7F.

**Fig. 4:**
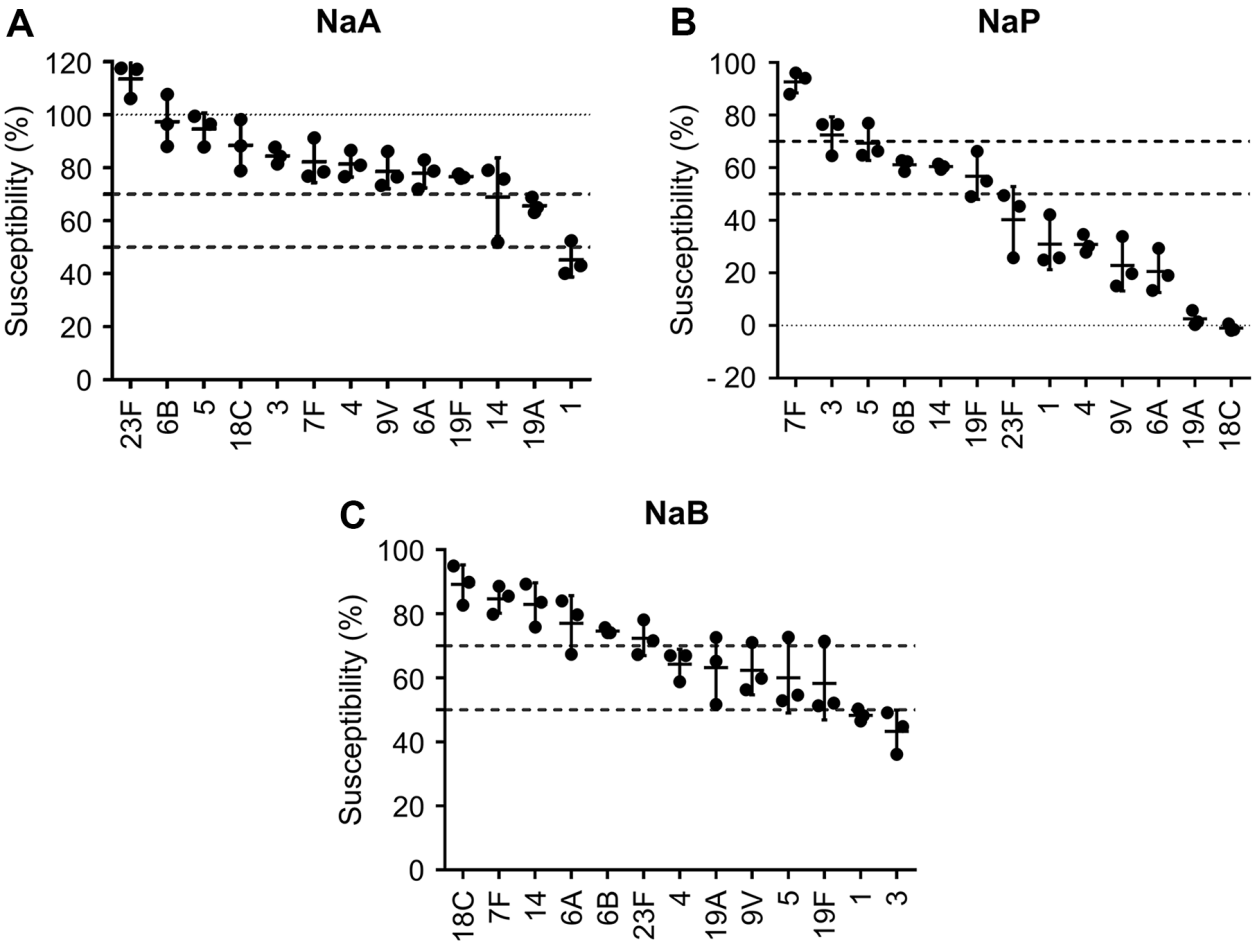
Growth inhibitory effects of SCFAs based on susceptibility of pneumococcal serotype. At maximum growth time point of each NT, the susceptibility was calculated as OD_600_ value of 100 mM (**A**) NaA, (**B**) NaP, and (**C**) NaB treated group divided by maximal OD_600_ value of each NT. Each dot refers to an experiment conducted independently and indicates the mean value ± standard deviation of triplicate samples. NaA, sodium acetate; NaP, sodium propionate; NaB, sodium butyrate.

The susceptibility of each serotype was determined through SCFA treatment at 100 mM (Fig. 4A-4C). When analyzing serotypes with 50% growth inhibition by SCFAs, acetate was seen only in serotype 1, butyrate in serotype 3, and propionate in serotypes 1, 4, 6A, 9V, 18C, 19A, and 23F, excluding 3 and 7F. When serotypes with 30% growth inhibition were analyzed with SCFAs, propionate showed inhibition against 11 serotypes of 13 serotypes tested, excluding 3 and 7F, and butyrate did against seven serotypes including 3. Acetate was only effective against 1 and 19A. Growth inhibition greater than 30% against serotype 7F was not seen with all SCFAs, but only acetate showed the highest inhibition at 17.8 ± 7.9%. Thus, we conclude that inhibition of pneumococcal growth by SCFAs is dependent on the serotypes.

### CPS Is Involved in Resistance to Growth Inhibition by SCFAs

**Fig. 5:**
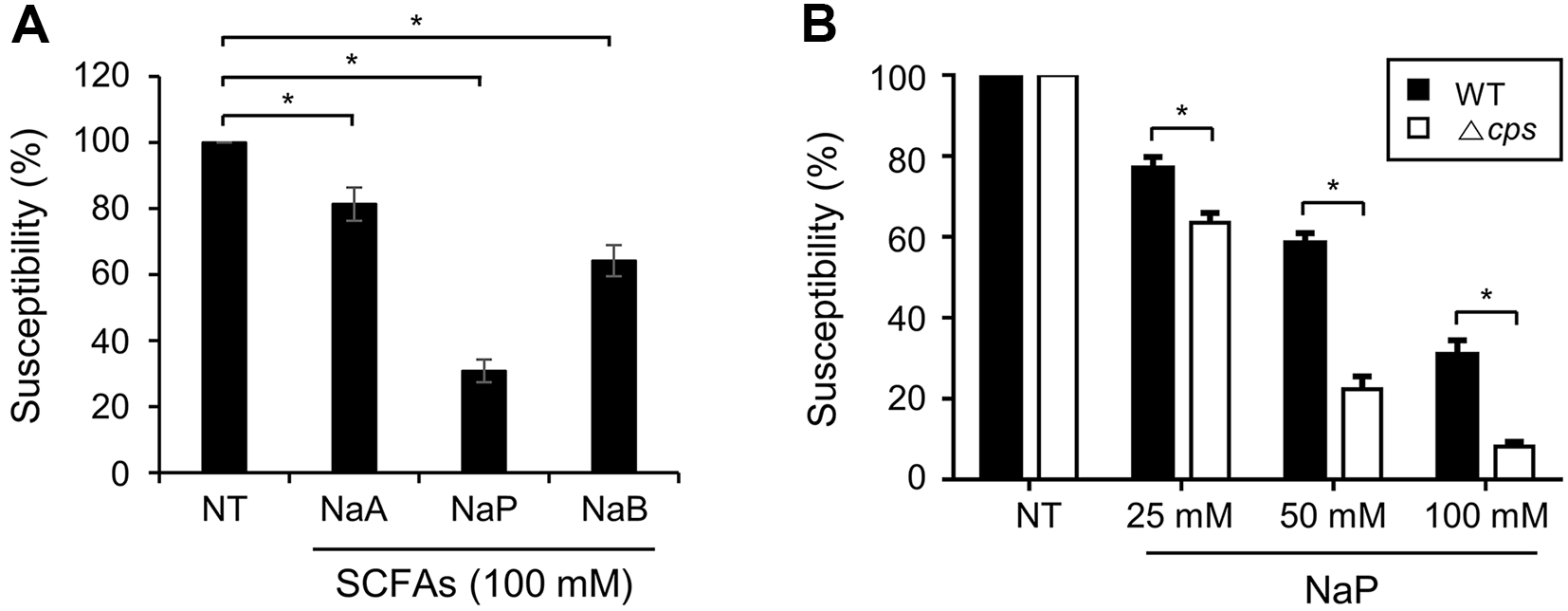
Effect of SCFAs on CPS-deficient *S. pneumoniae*. (**A**) CPS-deficient TIGR4 (Δ*cps*) was cultured in media with or without 100 mM SCFAs. Then, the susceptibility is calculated as OD_600_ value of 100 mM SCFAs treated group divided by maximum OD_600_ value of NT. (**B**) Wild type TIGR4 (WT) and Δ*cps* were cultured in media with or without various doses of NaP. The susceptibility is calculated as OD_600_ value of NaP treated group divided by maximum OD_600_ value of NT and compared to the susceptibility of WT strain. Data shown are representative of three separate experiments and indicate the mean value ± standard deviation of triplicate samples. Statistical significance was analyzed with Student’s *t*-test compared to NT or WT and among SCFAs treated groups (*, *P* < 0.05). NaA, sodium acetate; NaP, sodium propionate; NaB, sodium butyrate; NT, nontreatment.

The data above indicate that CPS type can interact with the inhibition of pneumococcal growth by SCFAs. Therefore, we compared the effect of SCFAs on the growth of nonencapsulated mutant (TIGR4Δ*cps*) and its parent strain (TIGR4). As for the results of TIGR4 shown above, capsule-deficient TIGR4 (TIGR4Δ*cps*) was also sensitive to propionate, moderately sensitive to butyrate, and less sensitive to acetate (Fig. 5A). When TIGR4Δ*cps* was incubated with the indicated concentration of propionate, the growth rate was significantly reduced compared to the parent strain (Fig. 5B). Furthermore, for both acetate and butyrate, the MIC of Δ*cps* was the same as the parent strain and required relatively high concentrations (500 mM) to reach MIC (Table 1). However, propionate required much lower concentrations to reach MIC (125 mM), and the MIC of Δ*cps* required an even lower concentration (62.5 mM). These results suggest that CPS provides resistance to the inhibitory action of propionate and that SCFAs can also affect nonencapsulated pneumococci.

**Fig. 6:**
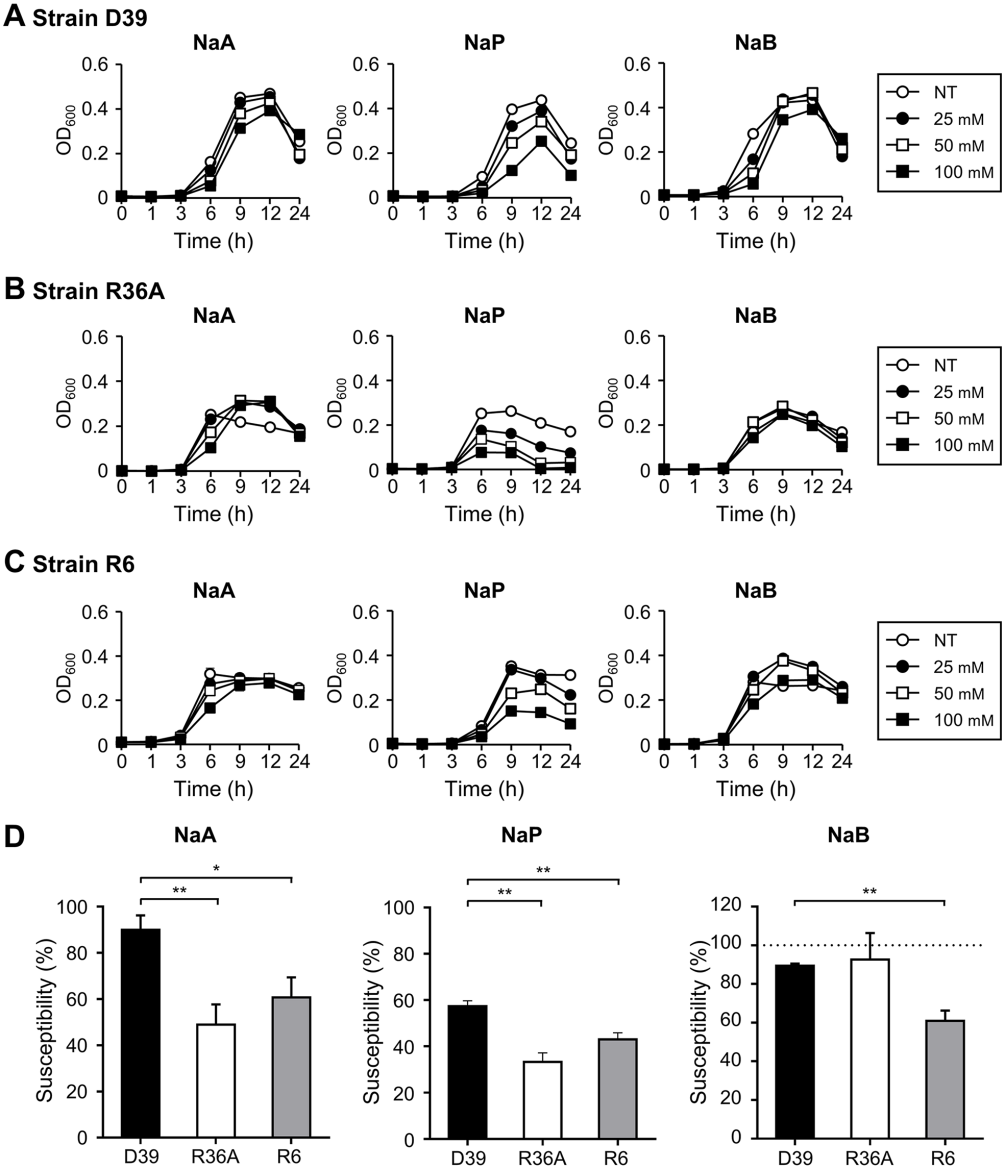
Growth inhibition of nonencapsulated *S. pneumoniae* by SCFAs. *S. pneumoniae* (**A**) D39, (**B**) R36A, and (**C**) R6, were cultured in media with or without various doses of SCFAs. The OD_600_ value was measured at 0, 1, 3, 6, 9, 12, or 24 h. (**D**) At maximum growth time point of each NT, the susceptibility is calculated as OD_600_ value of 100 mM SCFAs treated group divided by maximal OD_600_ value of each NT. Data shown are representative of three different experiments and indicate the mean value ± standard deviation of triplicate samples. Statistical significance was analyzed with Student’s *t*-test compared to NT or encapsulated strain (*, *P* < 0.05). NaA, sodium acetate; NaP, sodium propionate; NaB, sodium butyrate; NT, nontreatment.

Next, we compared the role of CPS in SCFA inhibition using two capsule-deficient strains (R36A and R6), which were derivatives of serotype 2 strain, D39. As shown in Fig. 6A-6C, growth of all three strains was drastically reduced by propionate, but not by acetate or butyrate. Of note, all capsule-deficient strains had significantly reduced viability compared to the parent strain (D39) (Fig. 6D). These data demonstrated that the susceptibility of pneumococci to SCFAs is dependent on the expression of CPS.

## Discussion

Despite the development of pneumococcal vaccines, pneumococcal infection remains rampant worldwide due to serotype switching and changes by PCV pressure [28]. The need for a new strategy to control infection is mostly due to the emergence of antimicrobial-resistant and/or non-vaccine serotypes [29, 30]. In this study, we investigated the anti-microbial effect of SCFAs on numerous pneumococcal serotypes to suggest new therapeutic strategies. Overall, SCFAs tended to inhibit the growth of PCV13 serotypes and even nonencapsulated strains. Among SCFAs, propionate most potently inhibited the growth of 11 serotypes, except serotypes 3 and 7F. Butyrate showed significant inhibition of serotype 3, but we have not been able to identify serotype 7F-targeting SCFAs.

The growth inhibitory effect of SCFAs, especially propionate, was also confirmed in other streptococci such as *S. gordonii* [21, 31]. For example, propionate can inhibit the growth of *S. gordonii* by regulating the gene expression associated with methionine synthesis [32]. In addition, other Gram-positive bacteria such as *S. aureus* and *E. faecalis* were inhibited by propionate [20, 33]. The effect of SCFAs was also confirmed in several pathogenic Gram-negative bacteria. Acetate inhibits the growth of *Escherichia coli* and propionate suppresses the growth of *S.* Typhimurium [11, 18, 34]. In addition, it has been reported that butyrate inhibits the growth of *Helicobacter pylori* [35]. Therefore, the inhibition of bacterial growth by SCFAs is dependent on the bacterial species, serotypes, and biochemical characteristics. In this study, we found that the inhibition of pneumococcal growth was serotype-specific, with propionate in particular being able to inhibit the growth of the most diverse serotypes. For example, serotype 7F was strongly resistant, whereas serotype 1 was susceptible to SCFAs. In addition, serotypes 5, 6B, 14, and 19F showed the susceptibility greater than 50% for all SCFAs tested. Thus, we infer that the type and ratio of SCFAs in the mucosa can affect pneumococcal colonization and infection.

There are hypotheses to explain how CPS confers resistance against propionate. First, CPS may serve as a physical barrier. *E. coli* resists complement-mediated killing by utilizing its capsule to decrease its permeability against the complement system [36]. Also, CPS may function as an electrochemical barrier. The partial negative charge of CPS generates repulsive force between propionate and CPS leading to reduced permeability [37]. Next, the physical properties of CPS, such as thickness and density, potentially provide protection. Serotypes 3 and 7F, associated with invasive disease, have well-preserved, dense, thick capsules [38-40]. We found that serotypes 3 and 7F were the most resistant serotypes against SCFAs. Therefore, *S. pneumoniae* presumably employs its CPS to resist against various physiological stressors including SCFAs through multiple protective mechanisms. Nonetheless, since *S. pneumoniae* can easily undergo its phase variation, which affects the thickness of capsules to avert the host’s innate immune response, further research to characterize the thickness of the CPS layer in each serotype is required [41, 42].

SCFAs produced in the gut contribute to the prevention of infections in various mucosal sites. In the gut, SCFAs control infection by directly inhibiting the growth and virulence of pathogenic bacteria. For instance, SCFAs reduce the motility of *Salmonella enterica* by lowering its intracellular pH and lessen pathogenicity by downregulating genes in pathogenicity islands [19, 43]. Furthermore, SCFAs alleviate the severity of colitis caused by *Shigella flexneri* through antimicrobial peptide production [44]. Since SCFAs produced in the gut can be absorbed and transferred into other organs, such as lung via bloodstream [45], the SCFAs can exert their anti-bacterial function at sites other than the intestine. In fact, orally administrated butyrate reduced severity of pulmonary tuberculosis by inhibiting the growth of *Mycobacterium tuberculosis* [46-48]. Collectively, these reports suggested a possibility that SCFAs produced in gut may suppress the pneumococcal growth in various organs by translocating into other body sites.

In summary, propionate was the most effective for inhibiting the growth of pneumococci, but it was not effective against 3 and 7F, which are highly expressed in capsules. However, butyrate was highly effective against serotype 3, and acetate was effective, albeit not significantly, against 7F. Therefore, appropriate combinations of three SCFAs can be used as a safe treatment to inhibit infection by various serotypes of pneumococcus. Since SCFAs are a biocompatible agent that exists even at high concentrations in the body, the addition of SCFAs for treatment can be useful and have less side-effects than other treatment strategies.

## Figures and Tables

**Table 1:** MIC of SCFAs to TIGR4 WT and Δ*cps*.

MIC^a^ (mM)	NaA^b^	NaP^c^	NaB^d^
TIGR4 WT	>500	125	>500
TIGR4 Δ*cps*^e^	>500	62.5	>500

^a^MIC, minimum inhibitory concentration; ^b^NaA, sodium acetate; ^c^NaP, sodium propionate; ^d^NaB, sodium butyrate; ^e^TIGR4 Δ*cps*, CPS-deficient TIGR4.
